# Mechanism of Action of Flavonoids of *Oxytropis falcata* on the Alleviation of Myocardial Ischemia–Reperfusion Injury

**DOI:** 10.3390/molecules27051706

**Published:** 2022-03-05

**Authors:** Yang Guo, Ben-Yin Zhang, Yan-Feng Peng, Leng Chee Chang, Zhan-Qiang Li, Xin-Xin Zhang, De-Jun Zhang

**Affiliations:** 1 Research Center for High Altitude Medicine, Key Laboratory of High-Altitude Medicine (Ministry of Education), Key Laboratory of Application and Foundation for High Altitude Medicine Research in Qinghai Province (Qinghai-Utah Joint Research Key Lab for High Altitude Medicine), Qinghai University, Xining 810001, China; 1910020117@qhu.edu.cn (Y.G.); 2014980001@qhu.edu.cn (Z.-Q.L.); 2 College of Eco-Environmental Engineering, Qinghai University, Xining 810016, China; benyinzhang@qhu.edu.cn (B.-Y.Z.); y200901j10538@qhu.edu.cn (Y.-F.P.); 3 Department of Pharmaceutical Sciences, Daniel K. Inouye College of Pharmacy, University of Hawai’i, Hilo, HI 96720, USA; lengchee@hawaii.edu; 4 School of Pharmacy, Xi’an Jiaotong Univeristy, Xining 710061, China; zhangxinxin360@mail.xjtu.edu.cn

**Keywords:** *Oxytropis falcata* Bunge, flavonoids, network pharmacology, myocardial ischemia reperfusion injury, JNK/p38MAPK/NF-κB

## Abstract

*Oxytropis falcata* Bunge is a plant used in traditional Tibetan medicine, with reported anti-inflammatory and antioxidants effects and alleviation of myocardial ischemia reperfusion injury (MIRI). However, the underlying mechanism against MIRI and the phytochemical composition of *O. falcata* are vague. One fraction named OFF1 with anti-MIRI activity was obtained from *O. falcata*, and the chemical constituents were identified by ultra-high-performance liquid chromatography coupled with tandem mass spectrometry (UHPLC–MS). The potential targets and signaling pathways involved in the action of *O. falcata* against MIRI were predicted by network pharmacology analysis, and its molecular mechanism on MIRI was determined by in vitro assays. The results revealed that flavonoids are the dominant constituents of OFF1. A total of 92 flavonoids reported in *O. falcata* targeted 213 potential MIRI-associated factors, including tumor necrosis factor (TNF), prostaglandin-endoperoxide synthase 2 (PTGS2), and the NF-κB signaling pathway. The in vitro assay on H9c2 cardiomyocytes subjected to hypoxia/reoxygenation injury confirmed that the flavonoids in OFF1 reduced myocardial marker levels, apoptotic rate, and the inflammatory response triggered by oxidative stress. Moreover, OFF1 attenuated MIRI by downregulating the ROS-mediated JNK/p38MAPK/NF-κB pathway. Collectively, these findings provide novel insights into the molecular mechanism of *O. falcata* in alleviating MIRI, being a potential therapeutic candidate.

## 1. Introduction

Ischemic heart disease is a highly prevalent threat worldwide, with a mortality rate of 16% [1]. Although the timely restoration of myocardial blood flow can reduce the size of the ischemic myocardium, revascularization may also aggravate the functional and structural impairment of the myocardium, thereby resulting in myocardial ischemia reperfusion injury (MIRI) [2,3]. Multiple therapeutic strategies have been applied to manage MIRI, including mechanical physiotherapy (e.g., ischemic pretreatment, ischemic post-treatment, and remote ischemic treatment) [4,5,6], and pharmacotherapy (e.g., adenosine, cyclosporine A and atrial natriuretic peptide) [7,8,9]. However, the efficacy of these approaches is limited. Traditional Chinese medicine (TCM) has been widely used in China for thousands of years, and it has better therapeutic effects on various complicated disorders with few side effects [10]. Numerous clinical and experimental studies have demonstrated that Chinese medical prescriptions and herbal medicine extracts may play a therapeutic role in MIRI by exerting an anti-inflammatory and anti-apoptotic effect, maintaining calcium homeostasis, providing the clearance of oxygen free radicals, and avoiding platelet aggregation [11,12,13,14]. In addition, TCM extracts or mixtures possess more effective pharmacological activities than purified compounds due to their multiple components and targets [15]. Thus, the cardioprotective effects of TCM may provide a therapeutic alternative for MIRI.

*Oxytropis falcata* Bunge, belonging to the Leguminosae family of perennial herbs, is widely distributed in the northwestern and southwestern regions of China, including Qinghai, Gansu, Tibet, Sichuan, and Xinjiang provinces [16]. As recorded in the Tibetan medicine book *Jingzhu Materia Medica*, the ability of *O. falcata* to promote blood circulation, relief pain, clearing of heat, and detoxification is well known [17,18,19]. Moreover, *O. falcata* is also included in many Chinese herbal formulas, such as *Qingpeng ointment*, *Shierwei Yishou San* and *Cheezheng Pain Relieving Plaster*, which have been applied in clinical practice in China for decades [20,21,22]. Currently, the compounds isolated and identified from *O. falcata* mainly include flavonoids, alkaloids, and saponins [18]. Furthermore, numerous studies revealed that flavonoids in *O. falcata* are the predominant active compounds responsible for the anti-inflammatory, anti-tumor, and antioxidant effects, as well as anti-cardiovascular diseases [17,23,24,25,26,27]. Therefore, the flavonoids of *O. falcata* are potentially correlated with the protective effects on MIRI. Extensive evidence revealed that flavonoids from other medical plants display diverse protective effects on MIRI. For instance, flavonoids from *Theobroma cacao* inhibit nitro-oxidative stress, inflammatory response, and apoptosis in MIRI by regulating AKT and NF-κB signaling pathways [28]. The total flavonoids from *Clinopodium chinense* alleviate myocardial injury through the modulation of the reactive oxygen species (ROS)-mediated Akt/Nrf2/HO-1 pathway in vivo and in vitro [29]. The flavonoids extracted from *Rosa rugosa* mitigate MIRI in mice, which is related with the inhibition of JNK and p38 MAPK signaling pathways [30]. Additionally, our previous report also demonstrated that *O. falcata* prevents cardiomyocyte injury induced by hypoxia/reoxygenation (H/R) in vitro and MIRI in vivo by reducing the levels of myocardial injury markers, inhibiting oxidative stress, as well as regulating the expression of apoptotic proteins [23,31,32,33]. However, to date, the detailed active compounds and molecular mechanisms of *O. falcata* involved in the amelioration of MIRI are still vague.

Unfortunately, TCMs are characterized by complex compositions and unclear mechanisms of action, which seriously hampers their globalization and modernization [34]. However, network pharmacology integrates bioinformatics, omics, pharmacology, and phytochemistry to elucidate the active compounds, multiple targets, synergistic interactions, and potential mechanisms of TCM [35,36,37]. In recent years, network pharmacology has helped researchers to successfully discover the pharmacological effects and mechanisms of action of Chinese herbal prescriptions (e.g., *Huo Tan Chu Shi* Decoction, *Shexiang Xintong* Ning, and *Baihe* Decoction) [38,39,40], Chinese medicinal herbs (e.g., Salvia miltiorrhiza, Huangqi, and Shanzha) [41,42,43], and ingredients of TCMs (e.g., curcumin, quercetin, and triterpenoids) [44,45,46].

In this study, ultra-high-performance liquid chromatography coupled with tandem mass spectrometry (UHPLC–MS) and network pharmacology were used to identify the potential bioactive components, hub node targets, and key putative pathways of *O. falcata* that mediate its therapeutic effect against MIRI. In addition, the cardioprotective effects of active fraction OFF1 from *O. falcata* were investigated in cardiomyocytes subjected to in vitro H/R injury.

## 2. Results

### 2.1. Effect of Different Fractions of O. falcata on H9c2 Cell Injury

H9c2 myoblasts, a cell model used as an alternative for cardiomyocytes, were treated with fractions from the *O. falcata* extract eluted with different ratios of petroleum ether and ethyl acetate from a silica gel column and then subjected to H/R to explore the fraction with anti-MIRI activity (Figure S1). The results showed that the fraction OFF1 eluted with petroleum ether and ethyl acetate (4:1) exerted a significant protective effect against H/R injury.

#### 2.1.1. Effects of OFF1 on Cell Viability

**Figure 1:**
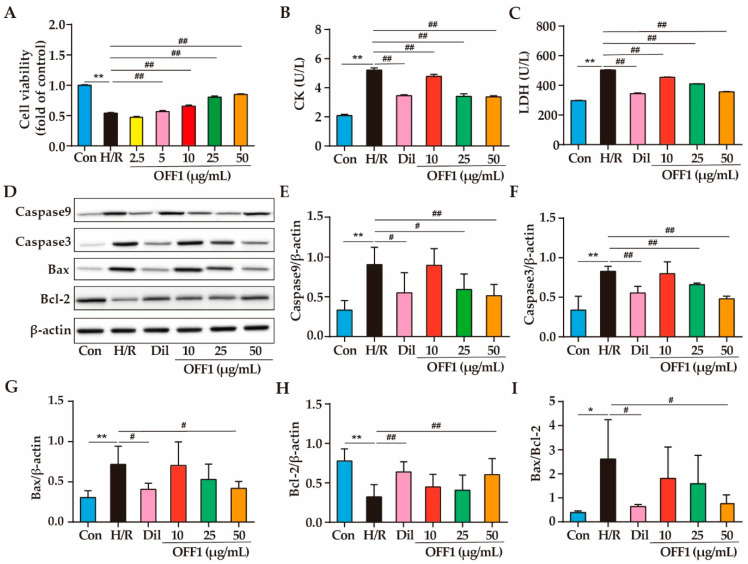
Effects of OFF1 on H9c2 cell injury. (**A**) Cell viability of H9C2 cells subjected to different treatments (*n*= 5). (**B**,**C**) Levels of cardiac markers under different treatments (*n* = 5). (**D**) Representative immunoblot showing the expression of Caspase9, Caspase3, Bax, and Bcl-2 (*n* = 3). (**E**–**I**) Semi-quantitative analyses of Caspase9, Caspase3, Bax, and Bcl-2 proteins (*n* = 3). Results are expressed as mean ± SD. * *p* < 0.05, ** *p* < 0.01 versus the control group; # *p* < 0.05, ## *p* < 0.01 versus the H/R group. Con, control group; H/R, hypoxia/reoxygenation; Dil, diltiazem; OFF1, prepared fraction from *O. falcata*.

H9c2 cells were pretreated with different concentrations of OFF1 and subjected to H/R; the cell viability of the H/R group markedly decreased by approximately 45.75% compared to the control group (Con) (*p* < 0.01; Figure 1A). However, the cell viability of the OFF1 (2.5, 5, 10, 25, and 50 μg/mL) groups was 1.03-fold, 1.14-fold, 1.43-fold, 1.76-fold, and 1.85-fold higher than that of the H/R group, respectively (*p* < 0.01; Figure 1A). The OFF1 pretreatment protected against cell death in a dose-dependent manner compared to the H/R group, and the dose of 50 μg/mL OFF1 was the most effective.

#### 2.1.2. Effects of OFF1 on Cardiomyocyte Damage

Creatine kinase (CK) and lactate dehydrogenase (LDH) are the markers identifying myocardial damage. CK activity in the cell supernatant of the Con, H/R, positive control diltiazem (Dil), and OFF1 (10, 25, and 50 μg/mL) groups were 2.08 ± 0.09, 5.21 ± 0.15, 3.46 ± 0.06, 4.77 ± 0.14, 3.40 ± 0.19, and 3.37 ± 0.07 (U/L), respectively. CK activity decreased approximately 33.59%, 8.45%, 34.74%, and 27.64% in the Dil and OFF1 groups, respectively, compared to the H/R group (*p* < 0.01) (Figure 1B). The levels of LDH in the cell supernatant of the Con, H/R, Dil, and OFF1 (10, 25, and 50 μg/mL) groups were 296.74 ± 2.33, 503.36 ± 1.80, 343.68 ± 4.91, 454.05 ± 1.96, 409.72 ± 1.11, and 355.69 ± 2.36, respectively. The LDH levels decreased approximately 31.72%, 9.80%, 18.60%, and 29.34% in these groups, respectively, compared to the H/R group (*p* < 0.01; Figure 1C).

#### 2.1.3. Effects of OFF1 on Cell Apoptosis

H/R dramatically increased the expression of pro-apoptotic proteins (Caspase9, Caspase3, Bax) as well as the Bax/Bcl-2 ratio and decreased that of anti-apoptotic protein (Bcl-2) (*p* < 0.05 or *p* < 0.01; Figure 1D). In contrast, the pretreatment with OFF1 and Dil protected against the effects of H/R injury on the apoptotic proteins (*p* < 0.05 or *p* < 0.01), indicating that the OFF1 fraction exerted a protective effect against H/R injury These results encouraged us to further investigate the chemical constituents and mechanism of action of OFF1. Taken together, OFF1 alleviates cell apoptosis in H/R-induced H9c2 cell injury.

### 2.2. Identification of the Main Components of OFF1

The chemical components of OFF1 were analyzed by UHPLC in the ESI negative and positive ion modes (Figure S2). Their accurate molecular formulas and molecular weight were identified by high-accuracy quasi-molecular ions ([M + H]^+^ and [M − H]^−^) with a mass error of 5 ppm, and qualitative analyses were mainly completed with MS/MS fragments. The results showed that OFF1 was mainly composed of flavonoids, and 31 of them including daidzein, 7-hydroxyflavone, maackiain, isoliquiritigenin, naringenin, and luteolin were tentatively identified, as shown in Table 1. Among them, Daidzein and maackiain belong to the isoflavone group; 7-hydroxyflavone, naringenin, and luteolin to the flavone group; and isoliquiritigenin to the chalcone group. Therefore, our next focus was primarily on flavonoids from *O. falcata*.

### 2.3. Network Pharmacology Analysis

#### 2.3.1. Selection of Active Compounds in *O. falcata*

A total of 115 flavonoids from *O. falcata* was found through the search of the relevant literature [18,19,23,27,43,47], and among them, 90 active compounds were screened out based on Lipinski’s Rule of five [48]. Myricetin and robinin were also taken into consideration due to their previously reported pharmacological effects [49,50] (Table S1). In total, 92 active compounds were used for the evaluation of the target prediction.

#### 2.3.2. Potential Targets and PPI Network Analysis

**Figure 2:**
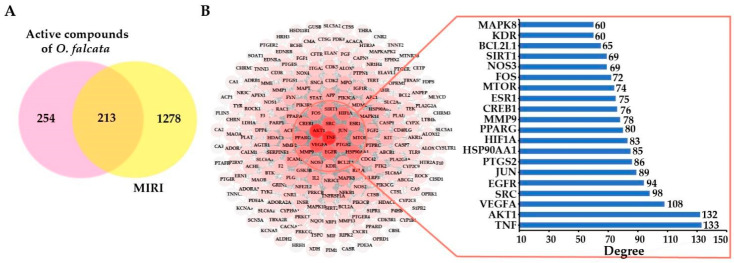
Screening of targets in MIRI. (**A**) Overlapping flavonoid-related and MIRI-related targets by Venn diagram. (**B**) Protein–protein interaction network and key crossover targets.

A total of 1167, 175, 638, and 178 MIRI-related targets was screened out from GeneCards, Disgnet, GeneCLip 3, and OMIM databases, respectively, and then 1491 targets were considered after removing the duplicates (Table S2). Subsequently, 131, 267, and 331 flavonoid-related targets were predicted from the databases of SwissTarget Prediction, SEA, and TargetNet, respectively, and 467 targets were considered after the removal of duplicate entries (Table S3). Finally, 213 overlapping targets between MIRI- and flavonoid-related targets were generated, which were likely targeted by flavonoids from *O. falcata* curing MIRI (Figure 2A, Table S4).

The PPI network was constructed by the STRING online platform in order to clarify the interaction among the intersection targets and screen out the most core targets. The PPI network consisted of 212 nodes and 2897 interactions (Figure 2B). Higher correlation coefficients (degrees) of interacting proteins were indicated by the stronger color intensity of the node. Network topological analysis identified TNF (degree = 133), AKT1 (degree = 132), VEGFA (degree = 108), SRC (degree = 98), EGFR (degree = 94), JUN (degree = 89), and PTGS2 (degree = 86) as the hub targets of flavonoids to protect against MIRI (Table S5).

#### 2.3.3. Compound-Disease-Target Network Analysis

**Figure 3:**
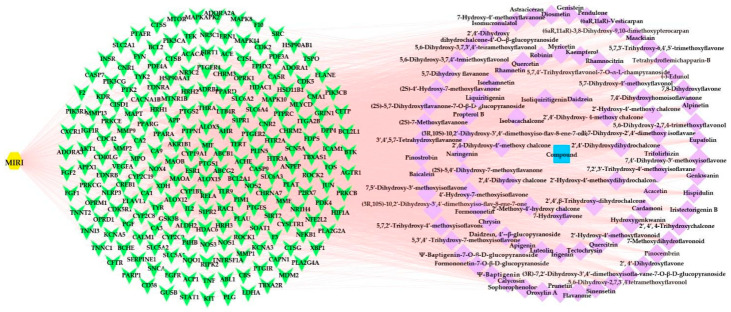
Active compound–MIRI–target network. The yellow hexagons indicate the disease, the green V indicate 213 targets, the purple squares indicate 92 active compounds, and the blue square indicates drug.

The compound-disease-target network was constructed to further evaluate the interaction and correlation between the potential targets of various flavonoids in *O. falcata* to protect against MIRI. This network consisted of 306 nodes and 4469 edges, and the average number of neighbors was 29.21. A total of 213 targets (green nodes) in MIRI (yellow node) corresponding to 92 active compounds (purple nodes) of *O. falcata* (blue node) was found, as shown in Figure 3. This result suggested that multiple compounds exerted synergistic effects against MIRI through multiple targets. The network analysis showed that 2′,4′-dihydroxy chalcone (degree = 97), isoliquiritigenin (degree = 94), 7-hydroxyflavone (degree = 89), daidzein (degree = 86), naringenin (degree = 53), and luteolin (degree = 51) might be the key flavonoids in the treatment of MIRI by *O. falcata* (Table S6).

#### 2.3.4. Functional Enrichment Analyses

**Figure 4:**
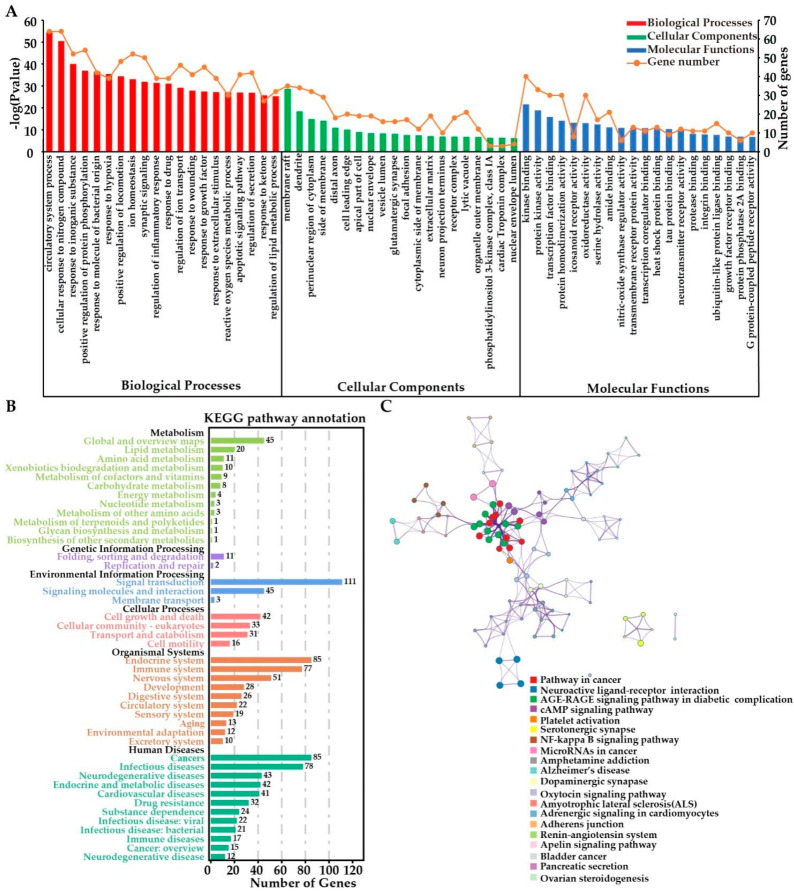
(**A**) GO enrichment analysis of the overlapping targets. (**B**) KEGG pathway annotation of the overlapping targets. (**C**) KEGG pathway analysis of the overlapping targets.

The GO enrichment analysis of the overlapping targets between flavonoids and MIRI was mainly categorized into the biological process (BP; 309 terms), cellular component (CC; 108 terms), and molecular function (MF; 118 terms) (Figure 4A and Tables S7–S9). As regards BP, flavonoids may prevent MIRI by their action on pathways involved in the circulatory system (GO:0003013; *p* < 0.001), cellular response to nitrogen compound (GO:1901699; *p* < 0.001), positive regulation of protein phosphorylation (GO:0001934; *p* < 0.001), response to hypoxia (GO:0001666; *p* < 0.001), regulation of inflammatory response (GO:0050727; *p* < 0.001), ROS metabolic process (GO:0072593; *p* < 0.001), and apoptotic signaling pathway (GO:0097190; *p* < 0.001) (Table S7). In addition, the significant enriched CC terms for the overlapping targets were membrane raft (GO:0045121, *p* < 0.001), perinuclear region of cytoplasm (GO:0048471; *p* < 0.001), side of membrane (GO:0098552; *p* < 0.001), distal axon (GO:0150034; *p* < 0.001), apical part of cell (GO:0045177; *p* < 0.001), and nuclear envelope (GO:0005635; *p* < 0.001). The significantly enriched MF terms included kinase binding (GO:0019900; *p* < 0.001), oxidoreductase activity (GO:0016491; *p* < 0.001), nitric-oxide synthase regulator activity (GO:0030235; *p* < 0.001), ubiquitin-like protein ligase binding (GO:0044389; *p* < 0.001), protein phosphatase 2A binding (GO:0051721; *p* < 0.001), growth factor receptor binding (GO:0070851; *p* < 0.001), and G protein-coupled peptide receptor activity (GO:0008528; *p* < 0.001).

KEGG pathway analysis (Figure 4B) further showed that the putative targets played a protective role in cardiovascular diseases by affecting lipid metabolism, signal transduction, and cell growth and death, as well as the immune and circulatory systems. The overlapping targets were assigned to 297 pathways; the top 20 significantly enriched pathways included energy metabolism, inflammation response, and cardiovascular-related pathways, for example the AGE-RAGE signaling pathway in diabetic complications (hsa04933; *p* < 0.001), cAMP signaling pathway (hsa04024; *p* < 0.001), platelet activation (hsa04611; *p* < 0.001), NF-κB signaling pathway (hsa04064; *p* < 0.001), adrenergic signaling in cardiomyocytes (hsa04261; *p* < 0.001), renin–angiotensin system (hsa04614; *p* < 0.001), and apelin signaling pathway (hsa04371; *p* < 0.001). Since the NF-κB pathway plays a pivotal role in preventing MIRI [28], our hypothesis is that flavonoids probably ameliorated MIRI through the regulation of ROS metabolic, anti-apoptotic, antioxidant, and anti-inflammatory effects.

### 2.4. Mechanism of Action of OFF1 in the Protection against MIRI In Vitro

#### 2.4.1. OFF1 Suppresses the Inflammatory Response

**Figure 5:**
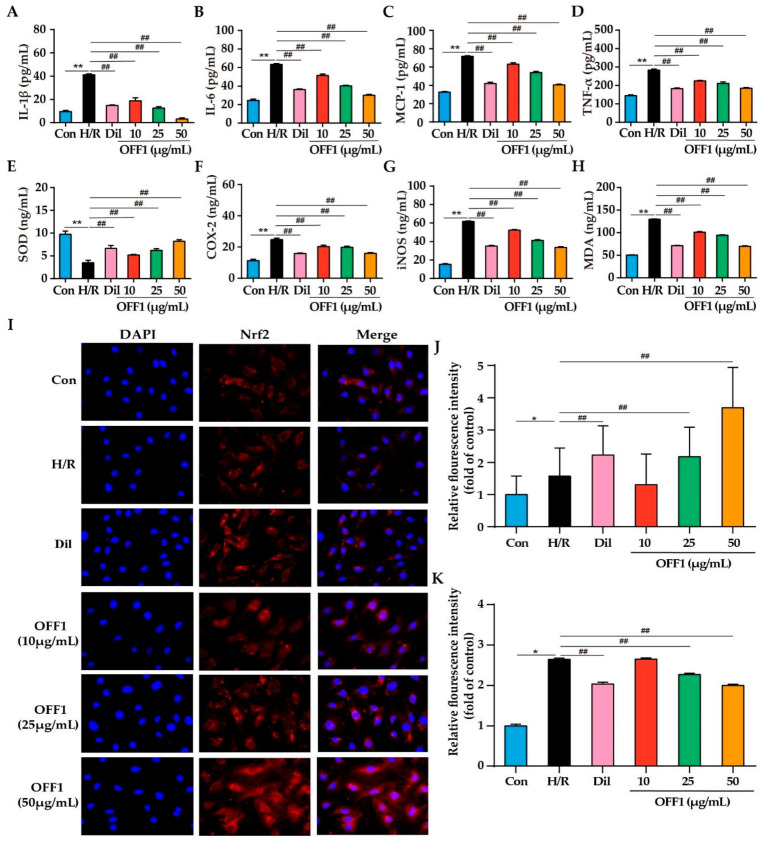
(**A**–**D**) Inflammatory cytokines levels in the indicated groups (*n* = 5). (**E**–**H**) Oxidative stress markers in the indicated groups (*n* = 5). (**I**,**J**) Representative images of immunostained cells showing the nuclear localization of Nrf2 (*n* = 3) (400x magnification). The blue fluorescence indicates the nucleus, and the red fluorescence indicates Nrf2. (**K**) ROS levels in the indicated groups (*n* = 5). Results are expressed as mean ± SD. * *p* < 0.05, ** *p* < 0.01 versus the control group; ## *p* < 0.01 versus the H/R group. Con, control group; H/R, hypoxia/reoxygenation; Dil, diltiazem; OFF1, prepared fraction from *O. falcata*.

The mechanism of action of OFF1 in the protection against MIRI was investigated in vitro according to the insights provided by the network pharmacology analysis. H9c2 cells subjected to H/R injury resulted in a significant increase in the cytokine levels of the pro-inflammatory interleukins (IL)-1β and IL-6, monocyte chemoattractant protein (MCP)-1, and tumor necrosis factor (TNF)-α in the supernatant of the cultured cardiomyocytes by 4.30-fold, 2.59-fold, 2.20-fold, and 1.96-fold, respectively, compared to the Con group (*p* < 0.01; Figure 5A–D). Conversely, IL-1β content was significantly reduced by approximately 64.08%, 54.57%, 69.49%, and 92.59% in the Dil and OFF1 (10, 25, and 50 μg/mL) groups compared with the H/R group, respectively (*p* < 0.01; Figure 5A). IL-6 concentrations in the Dil and OFF1 (10, 25, and 50 μg/mL) groups were lower by approximately 43.90%, 18.73%, 36.50%, and 52.45%, respectively, than those in the H/R group (*p* < 0.01; Figure 5B). MCP-1 levels in the Dil and OFF1 (10, 25, and 50 μg/mL) groups were lower than those in the H/R group by approximately 41.39%, 12.12%, 24.77%, and 43.38%, respectively (*p* < 0.01; Figure 5C). Moreover, TNF-α level was lower by 35.54%, 20.65%, 25.47%, and 34.89% in the Dil and OFF1 (10, 25, and 50 μg/mL) groups, respectively, when compared to the H/R group (*p* < 0.01; Figure 5D). These findings indicated that OFF1 alleviated the inflammatory response in H9c2 cells after H/R injury.

#### 2.4.2. OFF1 Suppresses Oxidative Stress

H9c2 cells subjected to H/R showed significantly reduced superoxide dismutase (SOD) levels by approximately 64.13% and increased levels of cyclooxygenase (COX-2), inducible nitric oxide synthase (iNOS), and malondialdehyde (MDA) by 2.20-fold, 4.03-fold, and 2.57-fold, respectively, as shown in Figure 5E–H. The SOD levels in the cell supernatant in the Dil and OFF1 (10, 25, and 50 μg/mL) groups were higher than those of the H/R group by approximately 1.91-fold, 1.48-fold, 1.78-fold, and 2.34-fold, respectively (*p* < 0.01; Figure 5E). COX-2 was lower by 36.00%, 18.02%, 19.54%, and 35.44% in the Dil and OFF1 (10, 25, and 50 μg/mL) groups, respectively, compared with the H/R group (*p* < 0.01; Figure 5F). iNOS content in the Dil and OFF1 (10, 25, and 50 μg/mL) groups was significantly decreased by approximately 42.96%, 15.26%, 33.21%, and 45.40%, respectively, compared with the H/R group (*p* < 0.01; Figure 5G). Additionally, the MDA concentration in the Dil and OFF1 (10, 25, and 50 μg/mL) groups were lower by approximately 45.06%, 27.29%, 22.09%, and 46.15%, respectively, than that in the H/R group (*p* < 0.01; Figure 5H). It is well established that ROS can trigger oxidative stress, inflammation response, and apoptosis in the myocardium, thus mediating MIRI or H/R injury [51]. Cells subjected to H/R showed significantly reduced ROS levels, which were attenuated by OFF1 or Dil in a dose-dependent manner (*p* < 0.01), as shown in Figure 5K. Collectively, these results revealed that OFF1 inhibited oxidative stress in the H9c2 cells after H/R injury.

#### 2.4.3. OFF1 Activates the Nrf2 Signaling Pathway

Previous studies showed that Nrf2 activation protects cardiomyocytes from oxidative stress-induced damage, and the level of intracellular ROS depends on the expression of the endogenous antioxidant Nrf2 [52,53]. Therefore, the ability of OFF1 to increase the expression of Nrf2 in H9c2 cells subjected to H/R injury was evaluated. OFF1 pretreatment significantly increased Nrf2 expression in the nuclei of the cardiomyocytes compared to the untreated H/R group (*p* < 0.01; Figure 5I,J). This result indicated that OFF1 could protect cardiomyocytes from oxidative injury by activating the Nrf2 signaling pathway.

#### 2.4.4. OFF1 Alleviates H/R Injury in Cardiomyocytes through the NF-κB Pathway

**Figure 6:**
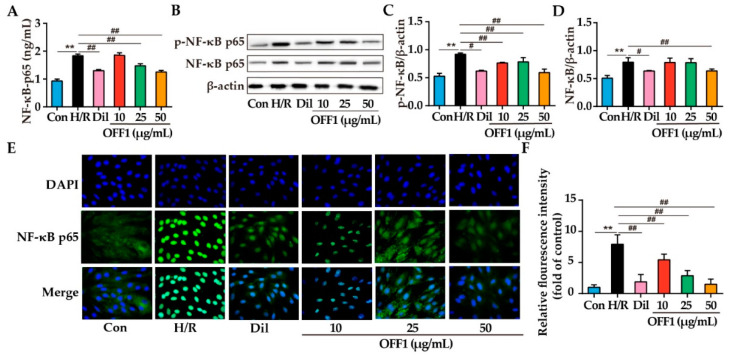
OFF1 alleviated H9c2 injury by the inhibition of the NF-κB pathway. (**A**) NF-κBp65 levels in the indicated groups (*n* = 5). (**B**) Representative immunoblots showing the expression of NF-κBp65 and IκB (*n* = 3). (**C**–**F**) Semi-quantitative analysis of NF-κBp65 and p-NF-κBp65 in the indicated groups. (**E**) Representative images of immunostained cells showing the nuclear localization of NF-κBp65 (*n* = 3) (400× magnification). (**F**) Relative fluorescence intensity of nuclear Nrf2. The blue fluorescence represents the nucleus, and the green fluorescence represents NF-κBp65. Results are expressed as mean ± SD. ** *p* < 0.01 versus the control group; # *p* < 0.05, ## *p* < 0.01 versus the H/R group. Con, control group; H/R, hypoxia/reoxygenation; Dil, diltiazem; OFF1, prepared fraction from *O. falcata*.

The NF-κB pathway was analyzed in the H9c2 cells subjected to H/R injury to further clarify the cardioprotective mechanism of OFF1. The levels of NF-κBp65 in the Con, H/R, Dil, and OFF1 (25 and 50 μg/mL) groups were 0.93 ± 0.07, 1.84 ± 0.06, 1.30 ± 0.04, 1.86 ± 0.08, 1.47 ± 0.07, and 1.25 ± 0.05 (U/L), respectively. The NF-κBp65 levels were decreased by approximately 29.18%, 19.71%, and 31.79% in the Dil and OFF1 (25 and 50 μg/mL) groups, respectively, when compared with the H/R group (*p* < 0.01; Figure 6A). The expression levels of NF-κBp65 and p-NF-κBp65 were significantly higher in the H/R group compared to the control group (*p* < 0.01) and decreased in cells pretreated with Dil and OFF1, as seen in Figure 6B–D. H/R stimulation promoted the nuclear translocation of NF-κBp65, which was suppressed by Dil and OFF1 (*p* < 0.01 or *p* < 0.01), as shown in Figure 6E,F. Hence, OFF1 could alleviate H9c2 cell injury induced by H/R by inhibiting the NF-κB signaling pathway.

#### 2.4.5. OFF1 Alleviates H/R Injury in Cardiomyocytes through the JNK/p38MAPK Pathway

**Figure 7:**
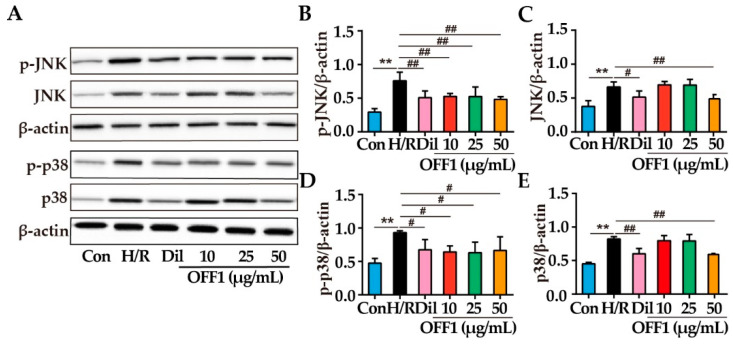
OFF1 attenuated H9c2 injury through the regulation of the MAPK pathway. (**A**) Representative immunoblot showing the expression of JNK and p38 (*n* = 3). (**B**–**E**) Semi-quantitative analysis of p-JNK, JNK, p-p38, and p38 in the indicated groups. Results are expressed as mean ± SD. ** *p* < 0.01 versus the control group; # *p* < 0.05, ## *p* < 0.01 versus the H/R group. Con, control group; H/R, hypoxia/reoxygenation; Dil, diltiazem; OFF1, prepared fraction from *O. falcata*.

Previous studies showed that JNK and p38MAPK are involved in the inhibition of NF-κB induced by different compounds [54,55]. The expression and phosphorylation of JNK and p38MAPK induced by OFF1 was examined to clarify whether JNK and p38MAPK are involved in the inhibition of NF-κB. H/R injury was associated with a significant increase in the expression of p-JNK, JNK, p-p38, and p38 (*p* < 0.01), and it was reduced by OFF1 (*p* < 0.05 or *p* < 0.01), as shown in Figure 7A–G. Dil exerted a similar effect on the MAPK pathway after H/R injury (*p* < 0.05 or *p* < 0.01). Taken together, OFF1 mitigated the effects of H/R injury on cardiomyocytes by inhibiting the JNK/p38 MAPK signaling.

## 3. Discussion

MIRI is a complex physiological and pathological process, mainly due to arterial bypass, thrombolytic therapy, and percutaneous coronary angioplasty in the recovery of cardiac tissue and organ blood flow [56]. In recent years, several studies have focused on the pharmacological and mechanical treatment of MIRI, although currently no approved therapy is available to improve the clinical outcomes and reduce the risk of MIRI [57,58]. TCMs have the advantages of high efficiency and low toxicity, thanks to their multiple targets [59]. *O. falcata* is used in Tibetan Medicine to treat several diseases, including leprosy, rheumatic pain, back pain, influenza, and tonsillitis [47]. Several anti-MIRI drugs have been developed from plant-derived natural compounds in recent years, exhibiting multiple pharmacological activities [60,61,62]. In addition, our previous studies found that the extracts of *O. falcata* have a protective effect on MIRI, although the specific mechanism was not clear [23,31,33]. Since the H9c2 cell line has similar membrane morphology and electrophysiological features as primary cardiomyocytes [63], it could be used as the optimal cell line for inducing H/R injury in vitro.

The present study evaluated a bioactive fraction of *O. falcata* called OFF1, which could protect H9c2 cell subjected to H/R from cell death, inhibit the release of CK and LDH, reduce apoptotic proteins such as Caspase9, Caspase3, and Bcl-2, as well as increase the levels of Bax and the ratio of Bax/Bcl-2. These findings demonstrated that OFF1 could protect H9c2 cell injury by suppressing cell apoptosis. The predominant component were flavonoids, including daidzein, 7-hydroxyflavone, maackiain, isoliquiritigenin, naringenin, and luteolin, which were identified by UHPLC–MS/MS analysis. Therefore, flavonoids probably exerted a crucial role in alleviating MIRI by *O. falcata*, accordingly providing a basis for the prediction of targets by network pharmacology analysis. Thus, the role of flavonoids of *O. falcata* were investigated. The analysis of network pharmacology revealed the intricate interactions among drug components, disease targets, signaling pathways, and molecular mechanisms of TCMs [11,63].

A total of 92 flavonoids of *O. falcata* was evaluated using Lipinsky’s RO5, and myricetin and robinin were additionally included due to their established pharmacological effects. For example, myricetin alleviates MIRI by up-regulating COX-2 and cytochrome P450 and p38, and downregulating fatty acid synthase antibody and glucose 6-phosphatedehydrogenase signaling pathway, thus reducing myocardial infarction, apoptosis, inflammation response, and oxidative stress [64]. Robinin reduces the levels of cardiac markers, antioxidant enzymes, lipid peroxidation products and inflammatory markers in cardiac tissue by regulating the TGF-β1 signaling pathway [65]. The major bioactive compounds (e.g., 2′,4′-dihydroxy chalcone, chrysin, isoliquiritigenin, 7-hydroxyflavone, daidzein, luteolin, and naringenin) of *O. falcata* were identified by the compound–target–disease network (Table S3). Although the active compounds did not clarify the predicted results (e.g., daidzein, 7-hydroxyflavone, maackiain, isoliquiritigenin, naringenin, and luteolin) of UHPLC–MS, the main flavonoids were consistent with the analysis. In this regard, our results were consistent with previous reports. For example, daidzein improves the left ventricular diastolic pressure and reduces inflammatory cytokines and apoptosis in a rat model of MIRI by regulating the NF-κB signaling pathway [66]. Luteolin markedly alleviates myocardial injury and cardiac dysfunction by activating the PI3K/Akt pathway and inhibiting the Siti1/NLRP3/NF-κB and ROS-mediated MAPK pathways [67,68]. In addition, naringenin protects against MIRI by regulating the NRF2/System xc-/GPX4 axis and cGMP-PKGIα signaling pathway [69,70]. Other compounds of *O. falcata* such as 7-hydroxyflavone and isoliquiritigenin also exert anti-inflammatory and antioxidant effects and can attenuate diabetic cardiomyopathy [71,72,73]. The putative targets in MIRI were also identified by PPI network analysis and included TNF, AKT1, VEGFA, SRC, EGFR, JUN, and PTGS2 as the core genes. Studies confirmed that TNF activates JNK and NF-κB by recruiting downstream signaling proteins, therefore aggravating MIRI [54,74]. The immunosuppressant azathioprine protects against MIRI by ameliorating oxidative stress, cell apoptosis, and inflammation by inhibiting the AKT1/GSK3β pathway [75]. VEGFA also plays a vital role in angiogenesis, vascular permeability, and inflammation [76]. Angiogenesis promotes collateral circulation around the ischemic tissue and restores myocardial blood flow, thereby greatly improving the efficacy and prognosis of patients with MIRI [77]. PTGS2, also known as COX-2, is activated in the early stage of inflammation, which is the key pathological mechanism of MIRI [78]. Consistent with the predicted results, OFF1 decreased TNF-α and COX-2 production in H9c2 cells subjected to H/R in vitro.

GO and KEGG analyses further indicated that the flavonoids of *O. falcata* could exert protective effects on cells subjected to MIRI by regulating the circulatory system, ROS metabolic process, inflammatory response, and cell apoptosis, which are correlated with the NF-κB signaling pathway. ROS broadly refers to oxygen-derived free radicals and non-free radicals; the over-production of ROS provokes the inflammatory response, exacerbates endotheliocytes dysfunction, and accelerates cell apoptosis, contributing to cardiac remodeling [79]. MIRI is associated with the overaccumulation of ROS, which promotes the apoptosis of cardiomyocytes by the inhibition of the MAPK signaling pathway [80], which lies upstream of the NF-κB and Nrf2 pathways [54,55]. Other studies also demonstrated that fullerol alleviates the inflammatory response by inhibiting ROS/p38MAPK/NF-κB and ROS/p38MAPK/FoxO1 pathways [81]. In the MIRI model of NADPH oxidase knockout mice, ROS production is reduced, and the myocardial infarction area is also reduced [82]. In the H9c2 cells injured by H/R, activation of the SIRT1/FOXO1/Mn-SOD antioxidant signaling pathway reduces H/R-induced ROS production, playing a protective role in cardiomyocytes [83]. Previous studies also showed that the activation of inflammatory factors in the MIRI process induces inflammatory reactions, resulting in local tissue degeneration and necrosis, accompanied by the destruction of the cell membrane, which further aggravates the necrosis and apoptosis of cardiomyocytes, leading to mitochondrial dysfunction [84]. Moreover, the inflammatory response caused by MIRI results in the continuous release of pro-inflammatory cytokines such as IL-6, TNF-α, ROS, and neutrophils. Neutrophils and ROS cause myocardial tissue damage through the lipid peroxidation of the cell membrane, protein denaturation, and DNA damage [85]. Notably, MIRI induces numerous death receptors such as apoptosis-related factors, tumor necrosis factors, and apoptosis-inducing ligands related to tumor necrosis factors. These receptors interact with the corresponding ligands to form death signal complexes, thereby inducing apoptosis [86].

**Figure 8:**
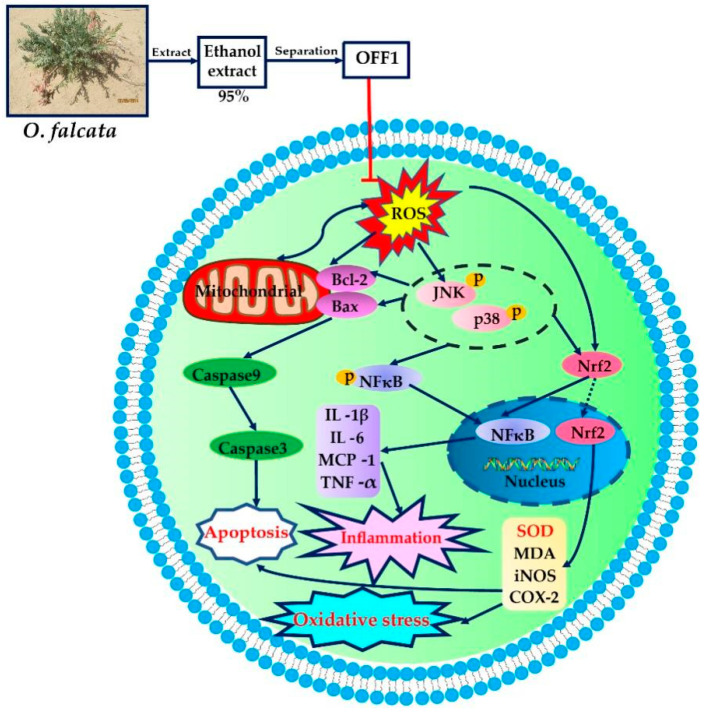
OFF1 protects cardiomyocytes from H/R injury by regulating the ROS-mediated JNK/p38MAPK/NF-κB pathway.

Therefore, the ROS metabolic process, inflammatory response, cell apoptosis, and NF-κB signaling pathway closely related to the pathogenesis of MIRI were evaluated in cell experiments to discover their upstream and downstream mechanisms. MAPK acts as an upstream regulator of NF-κB, which includes JNK and p38MAPK signaling pathways. The levels of inflammatory cytokines (e.g., IL-1β, IL-6, MCP-1, and TNF-α), oxidative stress-related cytokines (e.g., ROS, SOD, COX-2, iNOS, MDA, MCP-1, and Nrf2), and the relative expression and phosphorylation of NF-κBp65, JNK, and p38 were analyzed in the cardiomyocytes subjected to H/R injury. Our findings revealed that OFF1 protects cardiomyocytes from H/R-induced injury by inhibiting cell apoptosis, inflammation response, and oxidative stress by regulating the ROS-mediated JNK/p38MAPK/NF-κB pathway (Figure 8). These experimental findings were consistent with the predicted results and previous studies. For example, a study demonstrated that p38MAPK and JNK promote I/R, potentially playing an important role on MIRI [87]. The study of Liu et al. showed that mangiferin alleviates MIRI by inhibiting the MAPK/Nrf-2/HO-1/NF-κB pathway [54]. The protective mechanism of febuxostat in MIRI also depends on the inactivation of the MAPK/NF-κBp65/TNF-α signaling pathway [74]. NEAT1 aggravates MIRI by increasing the levels of myocardial enzymes, ROS, cardiomyocyte apoptosis, and inflammatory factors through the activation of the MAPK signaling pathway [88]. These findings showed that JNK, p38MAPK, and NF-κB could be potential targets in the treatment of MIRI by *O. falcata*, although multiple other signaling pathways and targets screened by the network pharmacology analysis need to be confirmed by functional studies.

## 4. Materials and Methods

### 4.1. Preparation of the Active Fraction of O. falcata

*O. falcata* was collected in 2015 from Gonghe County, Qinghai Province, China, and identified by professor Yongchang Yang, Northwest Plateau Institute of Biology. A voucher specimen of *O. falcata* was stored at College of Eco-Environmental Engineering, Qinghai University, as ZHANG2015-O18. One kilogram of the plant matter was ground into powder and extracted with 95% ethanol (10 L each time) at 75 °C under reflux for 3 h three times. The extracts were filtered, combined, and evaporated under reduced pressure to obtain a concentrated *O. falcata* extract [23]. The ethanol extract was resuspended in water and then extracted sequentially with petroleum ether and chloroform. The chloroform extract was subjected to a silica gel (Nano Micro Technology Co., Ltd., Suzhou, China) column (100–200 mesh) and eluted using a gradient system with petroleum ether and ethyl acetate (10:1 to 1:1). Then, 10 fractions were collected and concentrated by vacuum evaporator. These fractions were used for further pharmacological assays.

### 4.2. Identification of Main Compounds in Bioactive Fraction

We obtained an OFF1 of 4.6 g. The fraction OFF1 was selected and identified by ultra-high-performance liquid chromatography (thermoultimate 3000) coupled to mass spectrometry (UHPLC–MS, Thermo Scientific, Pleasanton, CA, USA) according to our previous report [23]. The chromatographic conditions were as follows: Hypersil GOLD aQ C18 (2.1 mm × 100 mm, 1.9 μm); column temperature: 40 °C; flow rate: 0.4 mL/min; mobile phase: acetonitrile (A) and water (B); gradient elution program: 2% A (0–2 min), 2–98% A (2–15 min). The mass spectrometric conditions were as follows: ion source: HESI-source (positive and negative ion mode); capillary temperature: 320 °C; auxiliary gas temperature: 350 °C; sheath gas: 40 arb; aux gas: 10 arb; spray voltage: 4.00 kV (+)/2.80 kV (−); resolution: 70,000 (full scan), 17,500 (dd-MS2); normalized collision energy: 20, 30, 40 Ev; scan range: 100–1500 m/z. We identified the main compounds in bioactive fraction by Predicted Compositions, mzCloud Search, MassList Search, mzVault Search and ChemSpider Search Library.

### 4.3. Identification of Active Flavonoid Compounds in O. falcata

The flavonoids in *O. falcata* were obtained from the relevant literature. The 2D structure of the flavonoids was drawn using Chemdraw 18.0, and the Smile structure was derived. Lipinski’s Rule of five (RO5), including molecular weight (MW) < 500, the number of hydrogen bond donors (nOHNH) < 5, number of hydrogen bond acceptors (nOH) < 10, lipid–water partition coefficient (miLogP) < 5, and number of rotatable keys (nRotb) ≤ 10 was applied to screen for flavonoids relevant to drug development (https://fafdrugs4.com, accessed on 15 April 2021). In addition, a few active compounds that did not meet the RO5 were included due to their high bioactivity.

### 4.4. Prediction of Active Compound-Related and MIRI-Related Targets

The compound-related targets in Homo sapiens were downloaded from the SwissTarget Prediction (http://www.swisstargetprediction.ch/, accessed on 15 April 2021), SEA (http://sea.bkslab.org, accessed on 15 April 2021), and TargetNet (http://targetnet.scbdd.com, accessed on 15 April 2021) databases. The criterion for the selection was the probability value (SwissTarget Prediction, accessed on 15 April 2021), Max Tc (SEA) or prob score (TargetNet) ≥ 0.4. MIRI-related targets were collected from GeneCards (https://www.genecards.org/, accessed on 15 April 2021), Disgnet (https://www.disgenet.org/search, accessed on 15 April 2021), GeneCLip 3 (http://ci.smu.edu.cn/genclip3/analysis.php, accessed on 15 April 2021), and OMIM (https://www.omim.org/, accessed on 15 April 2021) databases. UniprotKB (https://www.uniprot.org/) was used to standardize all targets to UniProt ID and gene name. The active compounds overlapping with MIRI-related targets were identified through Venn diagrams using Omicshare (https://www.omicshare.com/, accessed on 15 April 2021) to further evaluate the cardioprotective bioactive compounds.

### 4.5. Construction and Analysis of the PPI Network

The shared protein targets were incorporated into the STRING database (https://string-db.org/, accessed on 16 April 2021), and those with connection score 0.4 were used to construct the PPI network. Cytoscape 3.7.2 was used to visualize the PPI network. The hub targets were calculated by the Degree method of the Cytohubba plugin. Higher node degree was indicative of a stronger association among the target genes.

### 4.6. Construction of Drug–Compound–Gene–Disease Network

Cytoscape 3.7.2. was used to construct the “compound–gene–disease” network to determine the relationship between the drug (*O. falcata*), MIRI, active compounds, and potential targets. The network was analyzed to predict the hub active compounds of *O. falcata* against MIRI.

### 4.7. GO and KEGG Pathway Enrichment Analysis

GO and KEGG pathway analyses were performed using the metascape network tool (https://www.Metascape.com/, accessed on 17 April 2021) to clarify the biological function and pathways associated with the potential targets. The GO terms and KEGG pathways with a *p*-value < 0.05 were considered significantly enriched.

### 4.8. Cell Culture and H/R Modeling

H9c2 cells (Procell Life Science & Technology Co., Ltd., Shanghai, China) were cultured in Dulbecco’s modified Eagle’s medium (DMEM, 4.5 g/L glucose; GIBCO, San Diego, CA, USA) supplemented with 10% (*v*/*v*) fetal bovine serum (FBS; GIBCO, CA, USA) and 0.1% penicillin/streptomycin (Solarbio Science & Technology Co., Ltd., Beijing, China) and cultured at 37 °C under 5% CO_2_. The cells were used for different experiments once they reached 80% confluency. The cells were cultured in serum-free medium under hypoxic conditions consisting of 2% O_2_, 5% CO_2_, and 94% N_2_ for 12 h, and thereafter under normoxic conditions (95% air and 5% CO_2_) for 6 h for reoxygenation to simulate MIRI in vitro.

### 4.9. Cell Viability Assay

Cells were divided into control, H/R, and OFF1 groups. The cells were subjected to H/R pretreatment with different concentrations of OFF1 (2.5, 5, 10, 25 and 50 μg/mL) for 12 h, except for the control. A CCK8 assay kit (Elabscience Biotechnology Co., Ltd., Wuhan, China) was used to evaluate cell viability. Briefly, 10 μL CCK-8 and 100 μL DMEM were added to each well after H/R induction, and the cells were incubated for 1 h at 37 °C. The absorbance of each well was measured at 450 nm using a microplate reader (5200 Multi; Tanon Science and Technology Co. Ltd. Shanghai, China). After that, cells were divided into six groups: (a) control, (b) H/R, (c) H/R + Dil (20 μmol/L), (d) H/R + 10 μg/mL OFF1, (e) H/R + 25 μg/mL OFF1, and (f) H/R + 50 μg/mL OFF1.

### 4.10. Biochemical Assay

CK and LDH levels in the supernatants of H9c2 cells were detected using the corresponding kits (Jiancheng Bioengineering Institute, Nanjing, China), and the absorbance was measured at 450 nm. Commercial kits were also used to measure SOD, COX-2, and iNOS levels, as well as MDA activity (Jiancheng Institute of Biotechnology, Nanjing, China) according to the manufacturer’s protocols. IL-1β, IL-6, MCP-1, TNF-α, and NF-κBp65 levels were measured by ELISA using the corresponding kits (Jiancheng Bioengineering Institute, Nanjing, China) according to the manufacturer’s instructions.

### 4.11. Measurement of ROS Levels

Intracellular ROS levels were measured by the ROS Assay Kit (Solarbio Science & Technology Co., Ltd., Beijing, China) using the fluorescence indicator 2′,7′-dichlorodihydrofluorescein diacetate (DCFH-DA) probe [89]. Briefly, H9C2 cells were washed thrice with PBS and incubated with 10 μM DCFH-DA for 20 min at 37 °C in the dark. After washing thrice with PBS, the fluorescence intensity of the suspension was measured at the excitation wavelength of 88 nm and emission wavelength of 525 nm using a microplate reader (SpectraMax M5, San Diego, CA, USA). Image-Pro Plus 6.0 software (Media Cybernetics Inc, Rockville, MD, USA) was used to measure the mean fluorescence intensity.

### 4.12. Western Blotting

Total protein was extracted from H9c2 cells using RIPA lysis buffer and quantified with the BCA method (Biotek Biomedical Technology Co., Ltd., Beijing, China). The protein samples were resolved by SDS-PAGE and then transferred onto a PVDF membrane (Millipore, Boston, MA, USA). After blocking in 5% skim milk at room temperature for 1 h, the membrane was incubated overnight with antibodies targeting p-NFκBp65, NFκBp65, p-JNK, JNK, p38 (3031, 6956, 9255, 3708, and 8690, Cell Signaling Technology, Danvers, MA, USA), p-p38, Caspase-9, Caspase-3 (bs-0636r, bs-0049R, bs-0081R, Boosen Biotechnology Co., Ltd., Beijing, China), Bax (ab32503, Abcam, Cambridge, MA, USA), and Bcl-2 (AF6139, Affinity Biosciences, Cincinnati, OH, USA) at 4 °C. The following day, the membrane was washed thrice with TBST for 10 min each and incubated with the secondary antibody (MD912565, Biosco Biomedical Technology Co., Ltd., Beijing, China) for 1 h at room temperature. After washing thrice with TBST, the membrane was imaged using the ChemiDoc MP system (Bio-Rad Laboratories, Hercules, CA, USA). The grey scale values of the protein bands were quantified using ImagePro Plus 7.0 software, and β-actin was used as the internal reference.

### 4.13. Immunofluorescence

An immunofluorescence assay was performed as previously described. Briefly, the cell climbing slices were rinsed twice with PBS for 3 min each and fixed in 4% paraformaldehyde for 15 min. After washing again with PBS, the slices were blocked in PBS containing 5% goat serum for 1 h and incubated overnight with anti-Nrf2 (ab31163, Abcam, Cambridge, MA, USA) and anti-NF-kBp65 (ab16502, Abcam, Cambridge, MA, USA) antibodies in a humidified box at 4 °C. After the incubation with the secondary antibodies for 1 h, the cells were washed with PBS and counterstained with DAPI (Beyotime Institute of Biotechnology, Shanghai, China) for 5 min at room temperature. The slides were washed thrice with PBS for 5 min each and observed under a fluorescence microscope at 400x magnification. The average fluorescence intensity was measured using the Image-Pro Plus 6.0 software.

### 4.14. Statistical Analysis

Statistical analysis was performed using SPSS software 22.0 (IBM Corp., Armonk, NY, USA). The results were obtained from at least three independent experiments and indicated as mean ± standard deviation (SD). The comparison of multiple groups was performed by one-way analysis of variance. A value of *p* < 005 was considered statistically significant.

## 5. Conclusions

In this study, UHPLC–MS and network pharmacology were used to evaluate the multiple chemical components, multiple biological processes, and multiple signaling pathways involved in MIRI and in the effect of *O. falcata*, and the results were confirmed by in vitro experiments. *O. falcata* protected against MIRI by regulating inflammatory response, oxidative stress, and apoptosis by targeting the ROS mediated JNK/p38MAPK/NF-κB signaling pathway. These findings lay the foundation for the clinical application of *O. falcata* against MIRI.

## Figures and Tables

**Table 1:** Metabolites tentatively identified in OFF1 by UHPLC–MS in positive and negative ion modes.

No.	Name	Molecular Formula	Error (ppm)	MW (Measured)	ESI+/− (m/z)	MS/MS Fragment Ions
1	Daidzein	C_15_H_10_O_4_	1.77	254.0584	255.1022/253.1437	186.9758, 204.9867, 219.2112, 237.2218
2	Pinocembrin	C_15_H_12_O_4_	1.08	256.0738	257.0046/	215.9778, 256.2641, 210.9722, 233.0177
3	7-Hydroxyflavone	C_15_H_10_O_3_	2.16	238.06351	239.1283/238.8211	195.1230, 239.1073
4	Isoliquiritigenin	C_15_H12O_4_	1.44	256.07393	257.0046/–	192.9624, 210.9722, 215.9778, 233.0177
5	2,4,4′-Trihydroxydihydrochalcone	C_15_H_14_O_4_	−3.4	258.08833	–/257.1943	219.8445, 256.1910
6	Strobopinin	C_16_H_14_O_4_	−2.57	270.0885	–/269.2114	225.1019, 228.9878, 221.5644, 232.3254,
7	Sakuranetin	C_16_H_14_O_5_	2.24	286.08476	287.0556/–	121.0292, 153.0187, 165.0190
8	Formononetin	C_16_H_12_O_4_	1.02	268.0738	269.2116/267.0655	123.1166, 155.1437, 251.2016, 2215.1795
9	Bryaflavan	C_17_H_18_O_6_	0.58	318.1105	319.2024/–	163.1125, 219.1749
10	Luteolin	C_15_H_10_O_6_	−2.7	286.0470	–/285.9279	157.8609, 198.8836, 213.9066, 219.8449
11	Genistein	C_15_H_10_O_5_	2.36	270.0535	271.0971/269.2114	144.0830, 217.1947, 225.1028, 235.2047
12	Naringenin	C_15_H_12_O_5_	2.45	272.0691	273.0887/271.2271	200.9438, 205.9934, 215.1287, 245.0923,
13	Hispidulin	C_16_H_12_O_6_	−3.07	300.0625	–/299.2583	123.0438, 134.8927, 160.8404, 180.5528, 253.9687, 262.5875
14	Glycitein	C_16_H_12_O_5_	0.52	284.0686	285.0764/283.2633	166.9462, 192.9622, 214.9700, 210.9728
15	2′,4′-Dihydroxychalcone	C_15_H_12_O_3_	−3.4	240.0235	–/241.1943	219.8445, 256.1910
16	Miquelianin	C_21_H_18_O_13_	3.03	478.0762	479.0735/477.0154	303.0505, 229.0504, 153.0190
17	Isorhamnetin 3-glucuronide	C_22_H_20_O_13_	2.4	492.0916	493.3905/491.2122	153.0189, 302.0428, 317.0661
18	Lilaline	C_20_H_17_NO_7_	2.34	383.1014	384.1614/–	206.0816, 118.0656, 160.0769, 188.0707, 248.0921
19	Kaempferol 3-glucuronide	C_21_H_18_O_12_	−2.2	462.0788	–/461.0716	2253.0503, 261.9223, 320.8129, 357.7877
20	Maackiain	C_16_H_12_O_5_	0.42	284.0686	285.0764/–	214.9700, 192.9622, 210.9729, 184.9571
21	4’’’’-Acetylsagittatin A	C_34_H_40_O_19_	2.57	752.2183	–/751.2076	298.0475, 607.1785, 426.9629, 395.3199
22	Brosimacutin C	C_20_H_22_O_5_	−2.89	342.1457	–/341.1079	216.9878, 254.9855, 270.5942, 337.8138
23	Phloretin	C_15_H_14_O_5_	−2.51	274.0834	–/273.0159	134.4794, 146.6030, 169.0068, 179.8389
24	(S)-Equol	C_15_H_14_O_3_	1.78	242.0947	243.1915/–	111.0926, 180.1755, 228.1808, 107.0865
25	Isovolubilin	C_23_H_24_O_9_	−2.24	444.1410	–/443.1184	193.0493, 249.0610, 267.0711, 149.0591
26	Astragalin	C_21_H_20_O_11_	−2.37	448.0995	–/447.0060	78.9576, 148.9995, 179.0102, 96.9678
27	Rhamnetin	C_16_H_12_O_7_	1.65	316.0588	317.6330/–	167.0349, 243.0658,274.0481, 302.0421
28	Scrophulein	C_17_H_14_O_6_	1.71	314.07958	315.0506/–	287.0554, 231.0657, 259.0604, 203.0712
29	Isoquercetin	C_21_H_20_O_12_	−2.18	464.0945	–/463.0872	301.1073, 405.6862, 135.0440, 160.8422,
30	Isomucronulatol	C_17_H_18_O_5_	−2.03	302.0258	–/301.2122	113.0277, 120.0007, 181.0848
31	Rutin	C_27_H_30_O_16_	2.07	610.1547	611.1619/–	303.0504, 97.0289

## Data Availability

Processed data are contained within the article. Raw data are available from the corresponding author upon request.

## Supplementary Materials

The following supporting information are available online, Table S1: The active compounds in *O. falcata.* Table S2: The targets of myocardial ischemia reperfusion injury. Table S3: The targets of active compounds in *O. falcata.* Table S4: The overlapping targets of active compounds and MIRI. Table S5: PPI network analysis of overlapping targets. Table S6: The network analysis of drug-compound-disease-target network. Table S7: Biological process analyses of the overlapping targets. Table S8: Cellular component analyses of the overlapping targets. Table S9: Molecular function analysis of the overlapping targets. Table S10: KEGG pathway analyses of the overlapping targets. Figure S1: Effect of other fractions of *O. falcata* on H9c2 cell injury. Figure S2. The ESI negative and positive ion mode. Figure S3: The structure of major compounds.
