# Supplementary figures and images for: Mechanism of Action of Flavonoids of Oxytropis falcata on the Alleviation of Myocardial Ischemia–Reperfusion Injury
Source: Molecules. 2022 Mar 5;27(5):1706. doi: 10.3390/molecules27051706 (PMC8911915; doi:10.3390/molecules27051706)

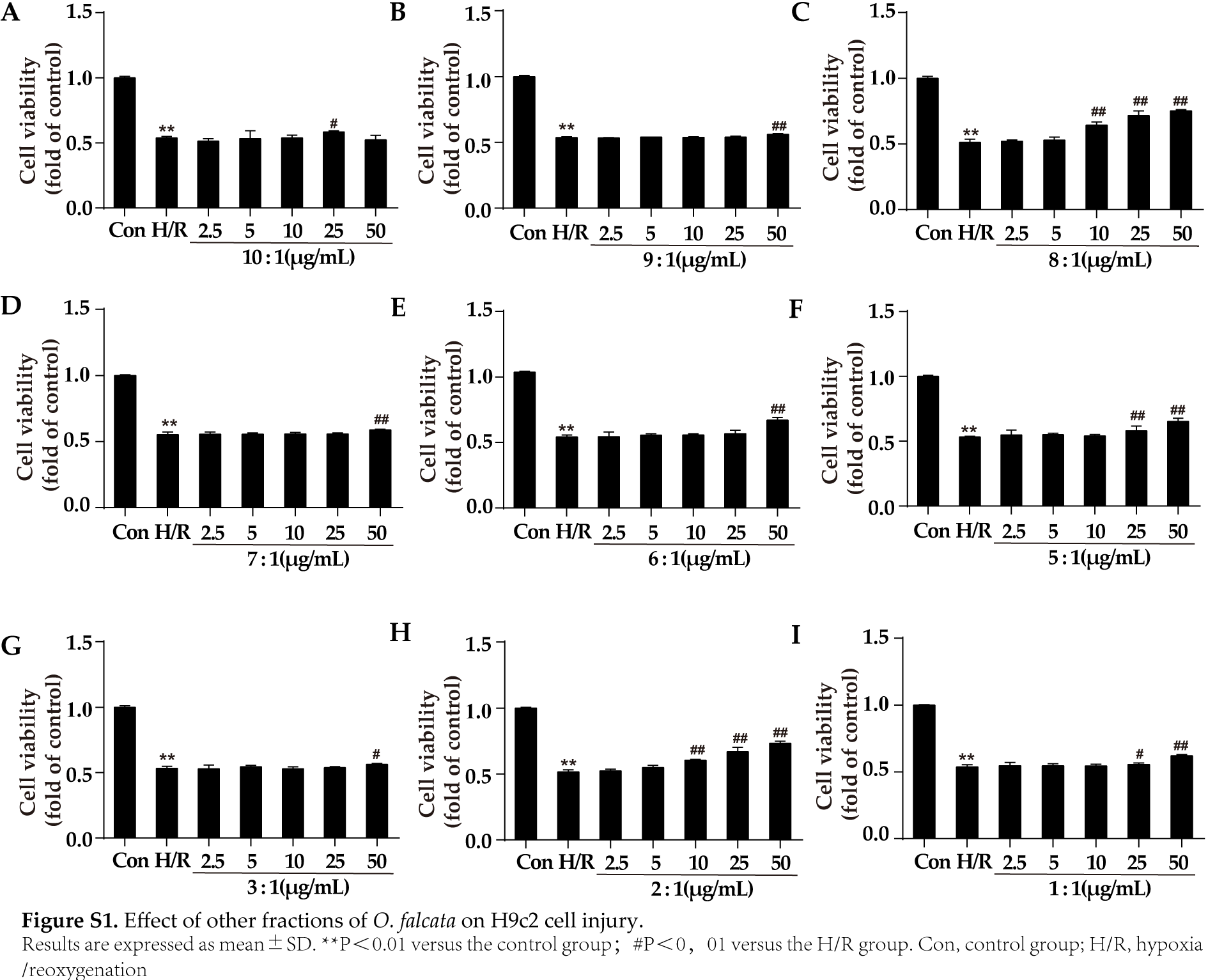

Supplement: Supplementary file 1 [file molecules-27-01706-s001.zip › Figure S1.tif]

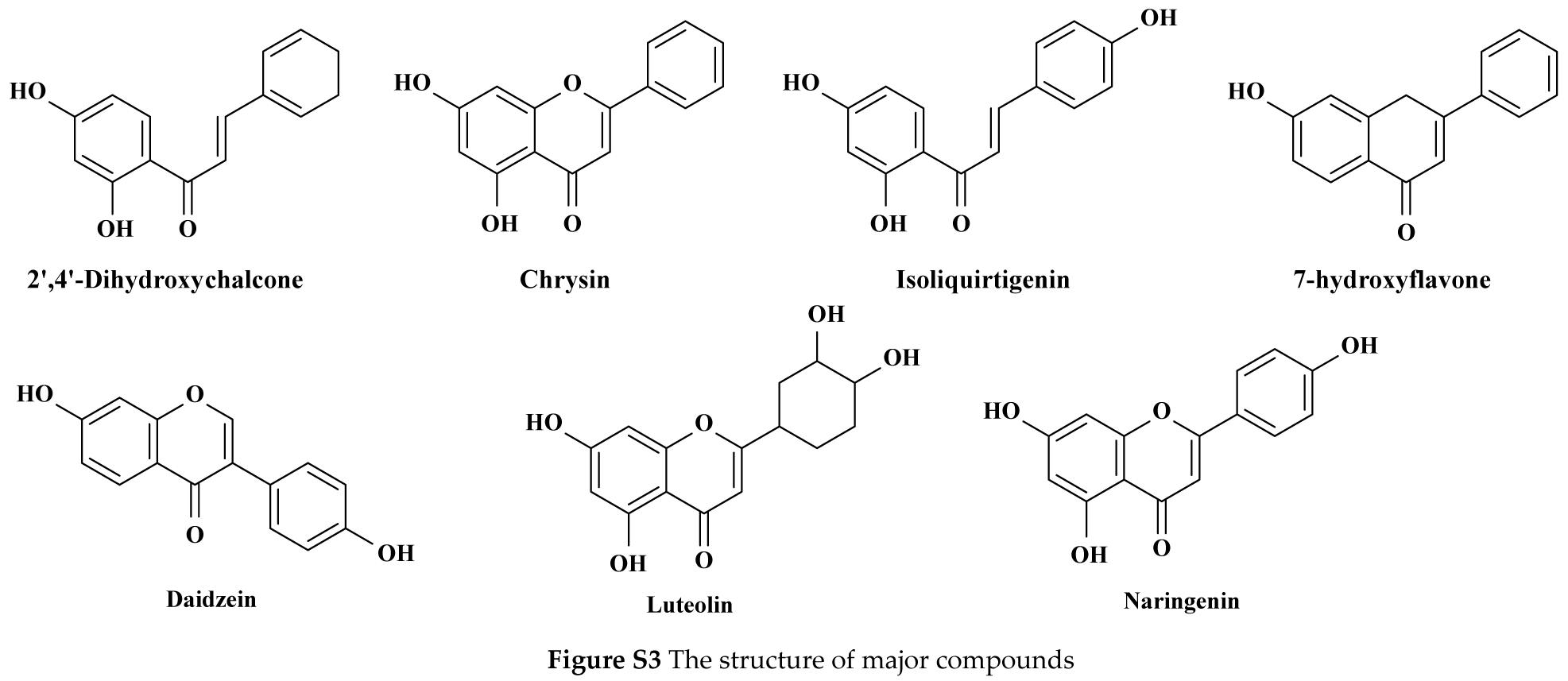

Supplement: Supplementary file 1 [file molecules-27-01706-s001.zip › Figure S3.tif]

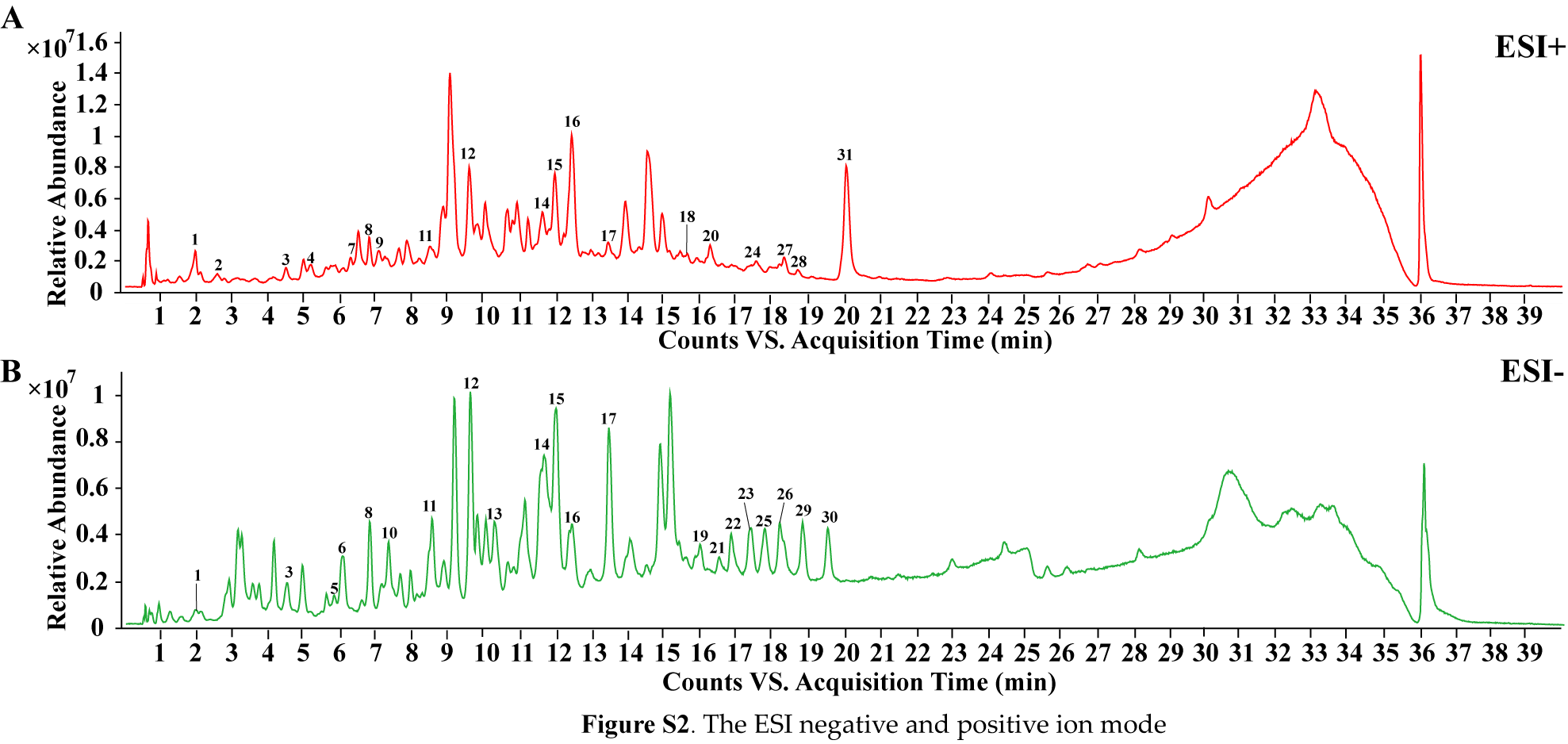

Supplement: Supplementary file 1 [file molecules-27-01706-s001.zip › FigureS2.tif]
